# Surgical Intervention in Neglected Ankle Fracture: A Case Report

**DOI:** 10.7759/cureus.26718

**Published:** 2022-07-10

**Authors:** Muhammed A Alsherbeeny, Mousa M Alhosawy, Mostafa S Almahe, Mohammed F Ali

**Affiliations:** 1 Orthopaedics and Trauma, King Salman Bin Abdulaziz Medical City, Almadinah Almunawara, SAU

**Keywords:** syndesmosis injury, bimalleolar fracture, malunion, neglected ankle fracture, ankle fracture

## Abstract

Ankle fracture is common in active young males. Treating ankle fractures can be straightforward or much more complicated; treatment options include nonoperative management or open anatomical reduction with rigid internal fixation. Successful treatment will allow early mobilization to avoid complications. Inadequate treatment, either nonoperative or operative management, may result in malunited ankle fractures. However, malunited ankle fractures due to the delayed presentation are very rare. An 18-year-old male presented to the clinic with a history of twisting injury to his right ankle two years ago. The patient sought medical advice once after injury, applied a back slab, and was advised for operative intervention. He refused the surgical intervention and was lost in follow-up. After two years, he presented again with ankle deformity and swelling. Assessment at initial presentation includes fibula malunion, medial malleolus malunion, and widening of the ankle mortise with talar tilt. Fogel and Morrey's performance index was used to evaluate the biomechanical result postoperatively*. *Delayed open anatomical reduction and rigid internal fixation of malunited ankle fractures to achieve normal ankle alignment will delay the onset of future degenerative changes and minimize the chance for early arthrodesis.

## Introduction

Ankle syndesmosis is a fibrous joint formed by the articulation between distal fibula and tibia; it is composed of both bony and ligamentous structures (anterior inferior tibiofibular ligament, posterior inferior tibiofibular ligament, intraosseous ligament, intraosseous membrane, Inferior transverse ligament), both are crucial for ankle stability [1]. The lateral ligaments group comprises three ligaments arising from the lateral malleolus and inserted into the talus (anterior and posterior talofibular ligaments) and the calcaneus (calcaneofibular ligament). The lateral ligament group act mainly to guard against the ankle over the inversion. Restoring normal anatomical syndesmosis is crucial to maintaining normal ankle function [1]. A normal anatomical syndesmosis is restored by maintaining fibular length and rotation. Malunited distal fibula fracture will lead to malunited syndesmosis [2]. Malunited syndesmosis can occur in nonoperative management and up to 50% with the surgical intervention [3].

Understanding the anatomy and morphology of medial malleolus is one of the essential steps in preoperative planning for proper fixation and avoiding postoperative complications [4]. The medial portion of ankle articulation also has bony and ligamentous components (superficial and deep deltoid ligament). The deltoid ligament arises from the medial malleolus and attaches to the talus, navicular, and calcaneus bones. Deltoid ligament guards against over-eversion of the ankle joint. Medial malleolus open reduction and internal fixation have great importance in the treatment of unstable ankle fracture; even fixation of isolated medial malleolus injury is recommended in cases of incongruent joint [5].

Treatment of ankle fractures is successful in most cases; failure of treatment will result in different pictures of malunion [6-7]. Malunited ankle fractures will affect the patient's day-to-day life activities due to pain, swelling, functional impairment, and development of joint arthrosis [8].

The timing of surgical intervention is usually determined by the condition of the soft tissue envelope around the ankle joint. Time of surgery is directly reflected in soft tissue complication and infection rate postoperatively [9]. Soft tissue edema increases in the first two days after injury, especially with high-energy trauma and severely displaced types of fractures [10]. Ankle swelling and skin blisters may delay surgical intervention up to 12 days after injury [9-10].

## Case presentation

An 18-year-old male presented with recurrent right ankle swelling, pain at the end of the day, and deformity. He had an ankle fracture two years earlier, which was treated in a back slab for two weeks. The patient removed the slab and refused surgical intervention at the time of injury. He came again as the pain and deformity interfered with his daily life activities, and he couldn't wear a shoe. His past medical and surgical history was irrelevant.

Clinical examination relieved walking on the medial ray of the foot with an eversion position. There was decreased active and passive ankle dorsiflexion and plantar flexion with severe reduction of ankle eversion and inversion. There was no skin abnormality or callosities.

**Figure 1:**
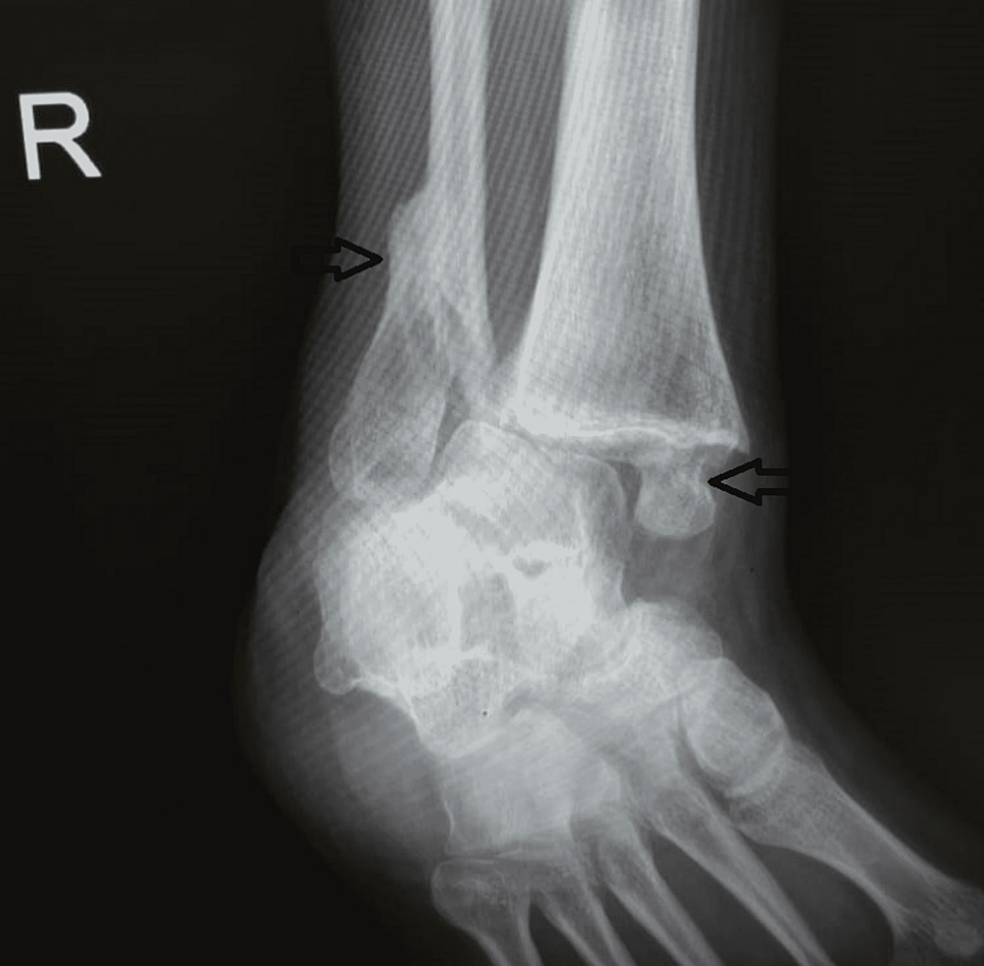
Preoperative plain radiograph anterior-posterior (AP) view showed the site of malunion

**Figure 2:**
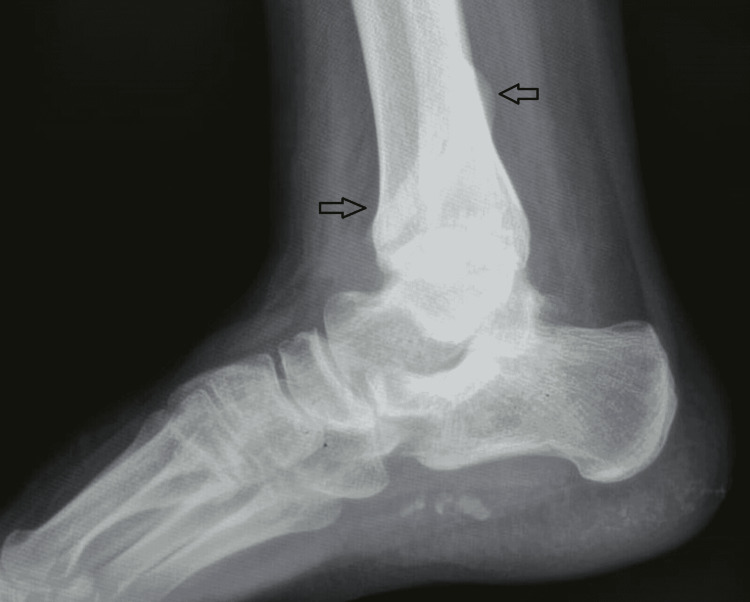
Preoperative radiograph lateral view showed lateral malleolus malunion

**Figure 3:**
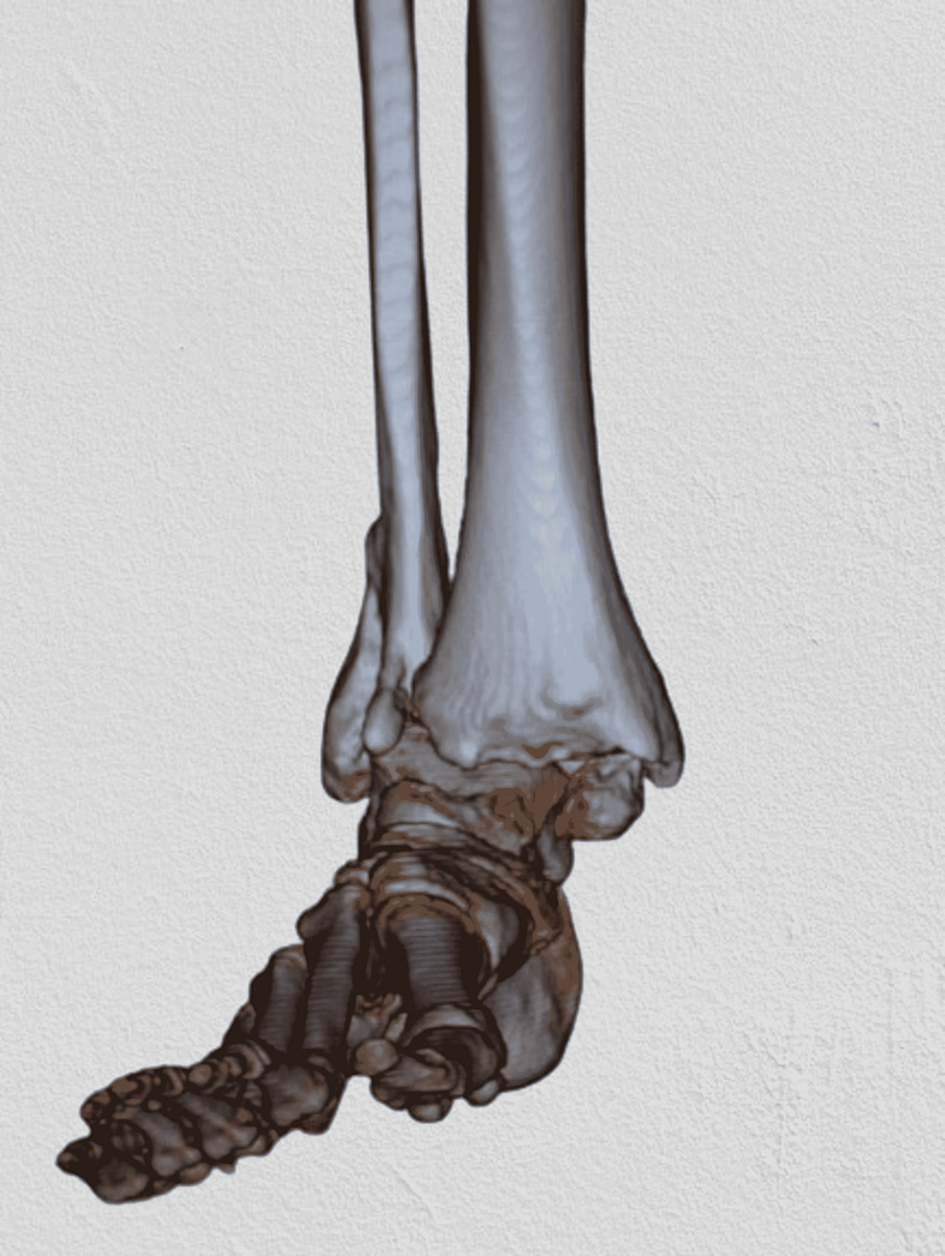
CT scan of the right ankle

**Figure 4:**
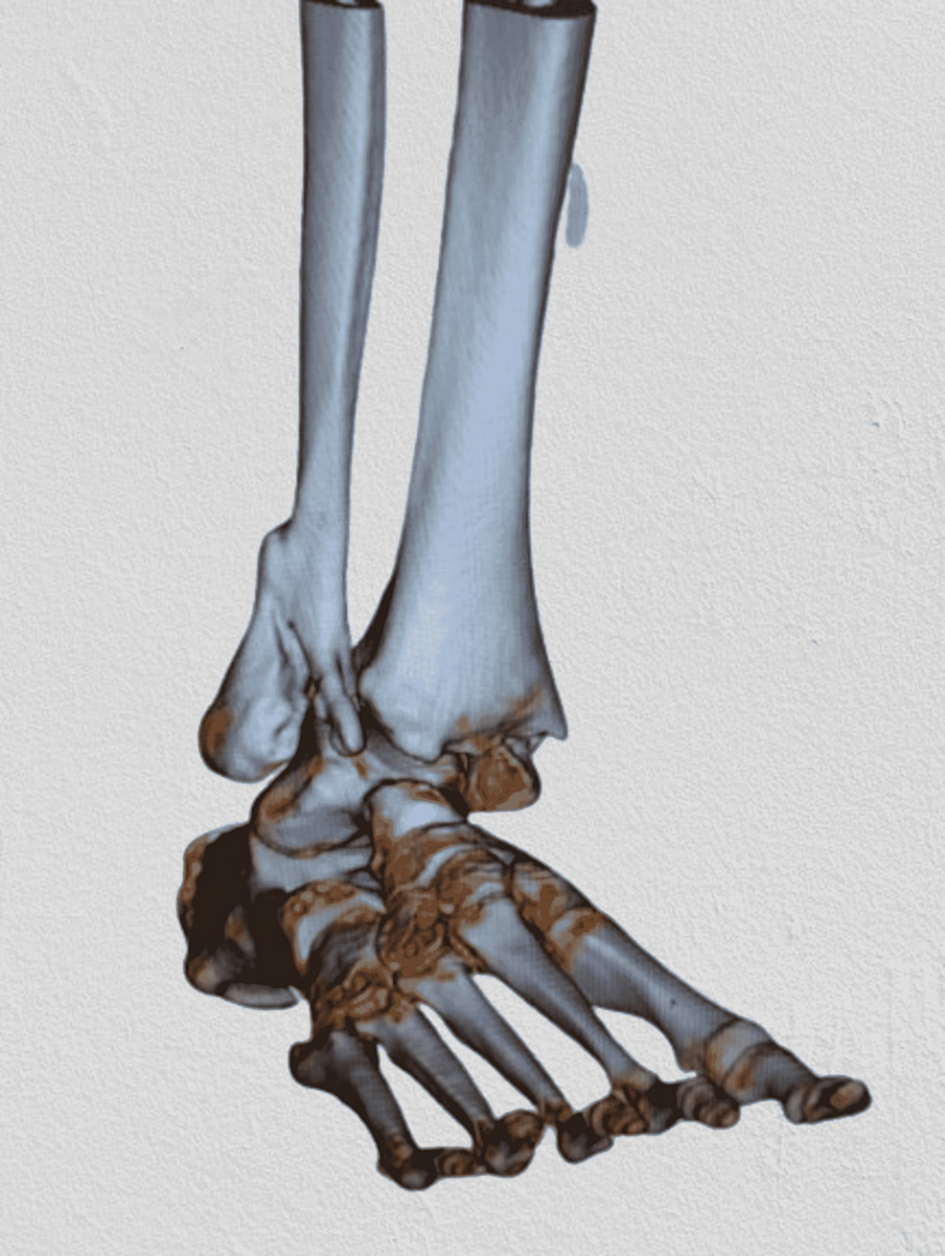
CT scan of the right ankle

Plain radiographs (Figures 1-2) showed a severely displaced malunited ankle fracture. There was shortening, rotation, and loss of alignment of distal fibula with evidence of syndesmotic injury. Also, non-united medial malleolus fracture with severe rotation and lateral displacement was evident. Talar tilt with no radiological evidence of arthritic changes at the tibiotalar and subtalar joint was seen. CT scan (Figures 3-4) confirmed the data obtained from plain radiographs and relieved no changes at the plafond and talus articular surfaces.

Surgical procedure

The patient was in a supine position. First, medial malleolus osteotomy was performed with the medial clear space debridement through a direct medial approach. The deltoid ligament was intact. The medial approach was planned first as the malunited laterally displaced medial malleolus interfered with the reduction of talus and restoration of normal fibular alignment.

The old malunited fracture site was identified with a direct lateral approach to the lateral malleolus and distal fibula. The callus was removed gently to avoid fracture of the fibula. The distal tibiofibular joint was debrided to free the distal fibula, which allows restoration of normal alignment.** **An osteotomy at the old fracture site was done.

Preliminary fixation of medial malleolus was done with K-wires; we tested reduction of the talus and checked the congruity of the ankle joint. Anatomical reduction of the lateral malleolus was done with the restoration of length and rotation using a one-third tubular plate. Fixation of the medial malleolus with screws was performed next. Lastly, the syndesmotic screws were inserted.

Rehabilitation and evaluation

**Figure 5:**
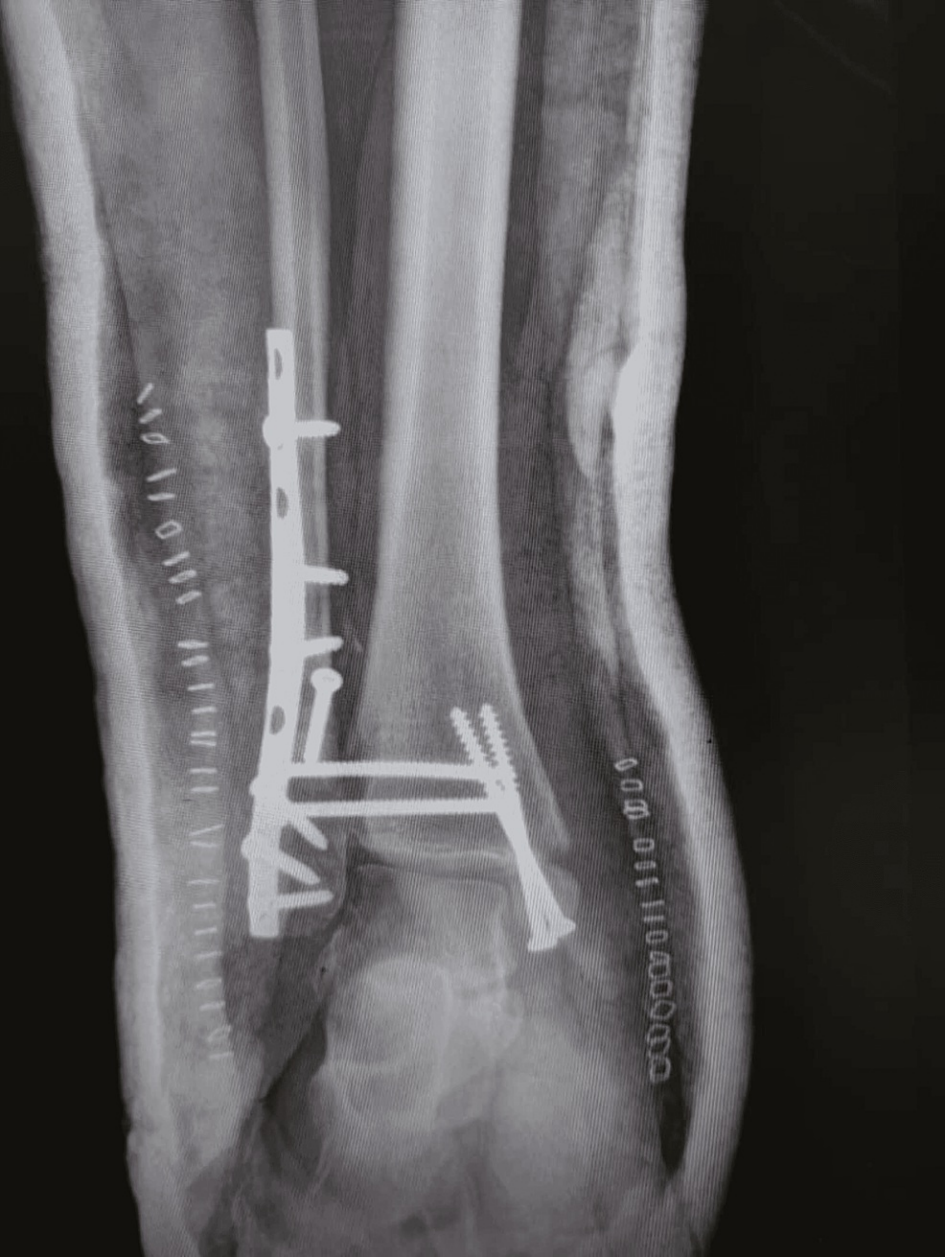
Postoperative radiograph anterior-posterior (AP) view

**Figure 6:**
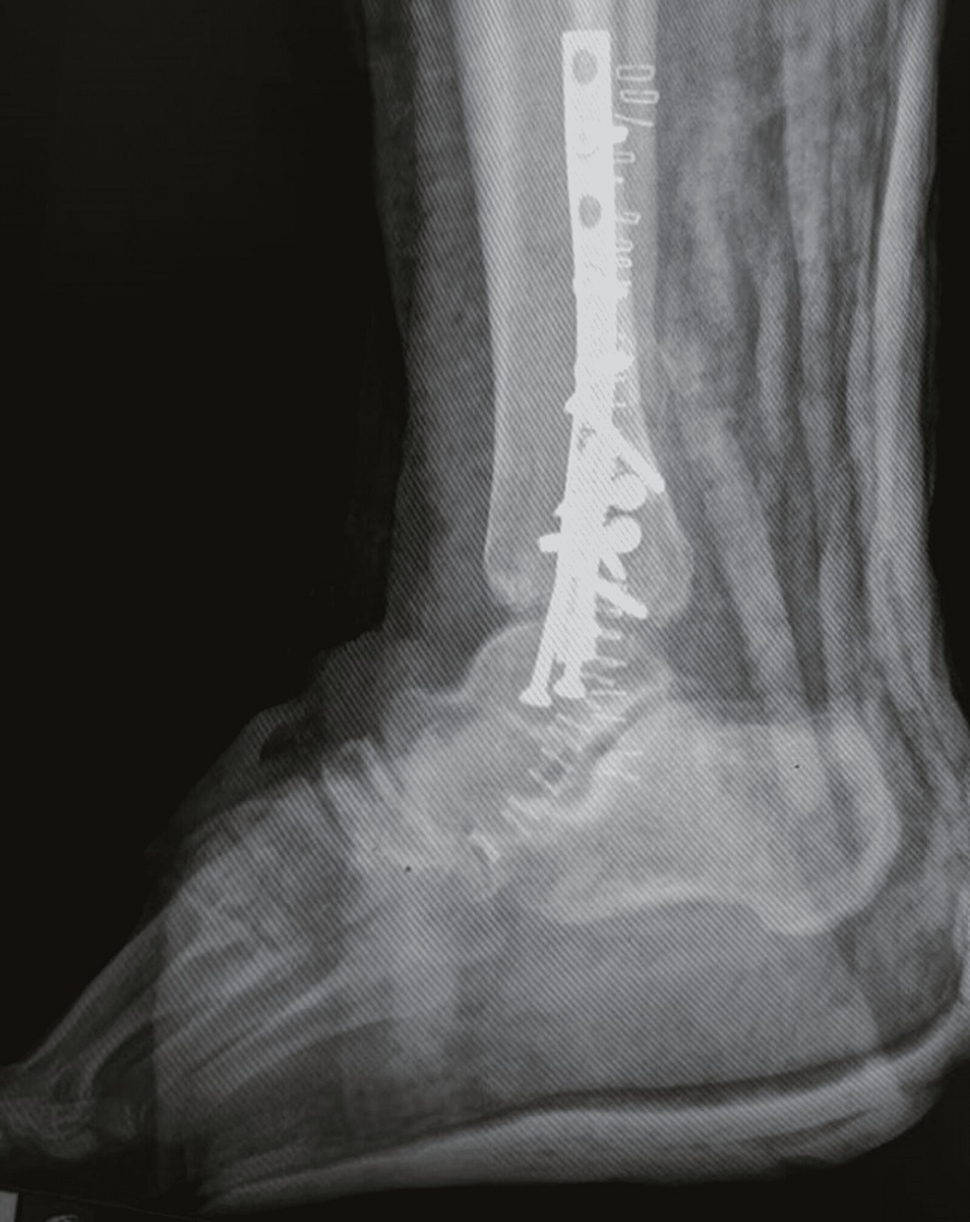
Postoperative radiograph lateral view

Immediately after the operation limb was elevated with ice application to decrease soft tissue edema. Plain radiographs were obtained postoperatively, which showed anatomical alignment with congruent joints (Figures 5-6). The patient was discharged after two days in a back slab. Three weeks after the operation, sutures were removed, and physiotherapy was started. The outcome was evaluated using the visual analog pain scale and Fogel and Morrey performance index. The ankle and subtalar joint movement was assessed as normal, limited, or absent. Follow-up showed favorable recovery with improvement in all objective parameters. The pain was absent in the second postoperative month. The Fogel and Morrey performance index was more than 76 at 24 postoperative months. The patient clinical examination of the ankle was normal without any morbidity. Ankle dorsiflexion and plantarflexion were normal, with a normal gait pattern.

**Figure 7:**
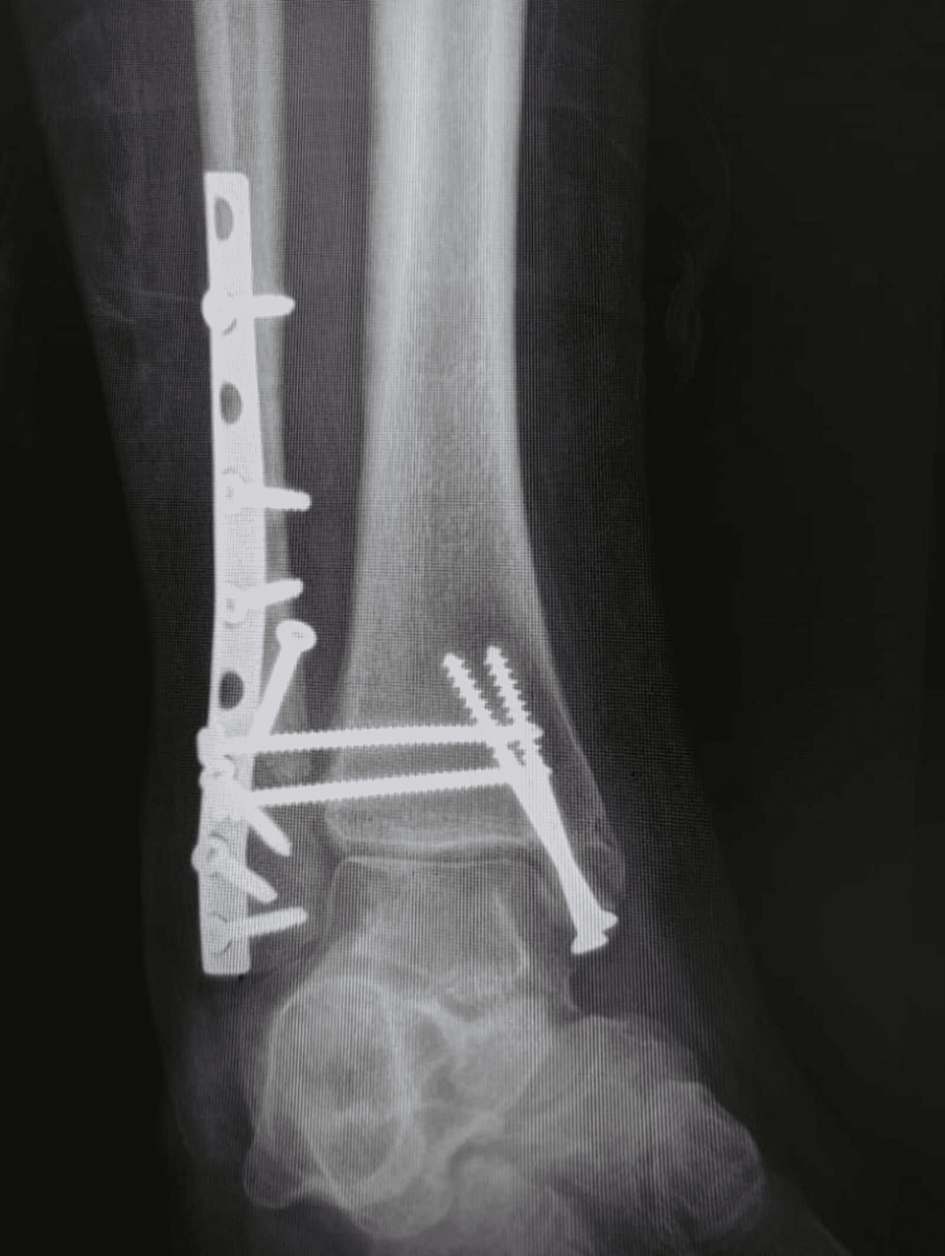
Follow-up radiograph anterior-posterior (AP) view

**Figure 8:**
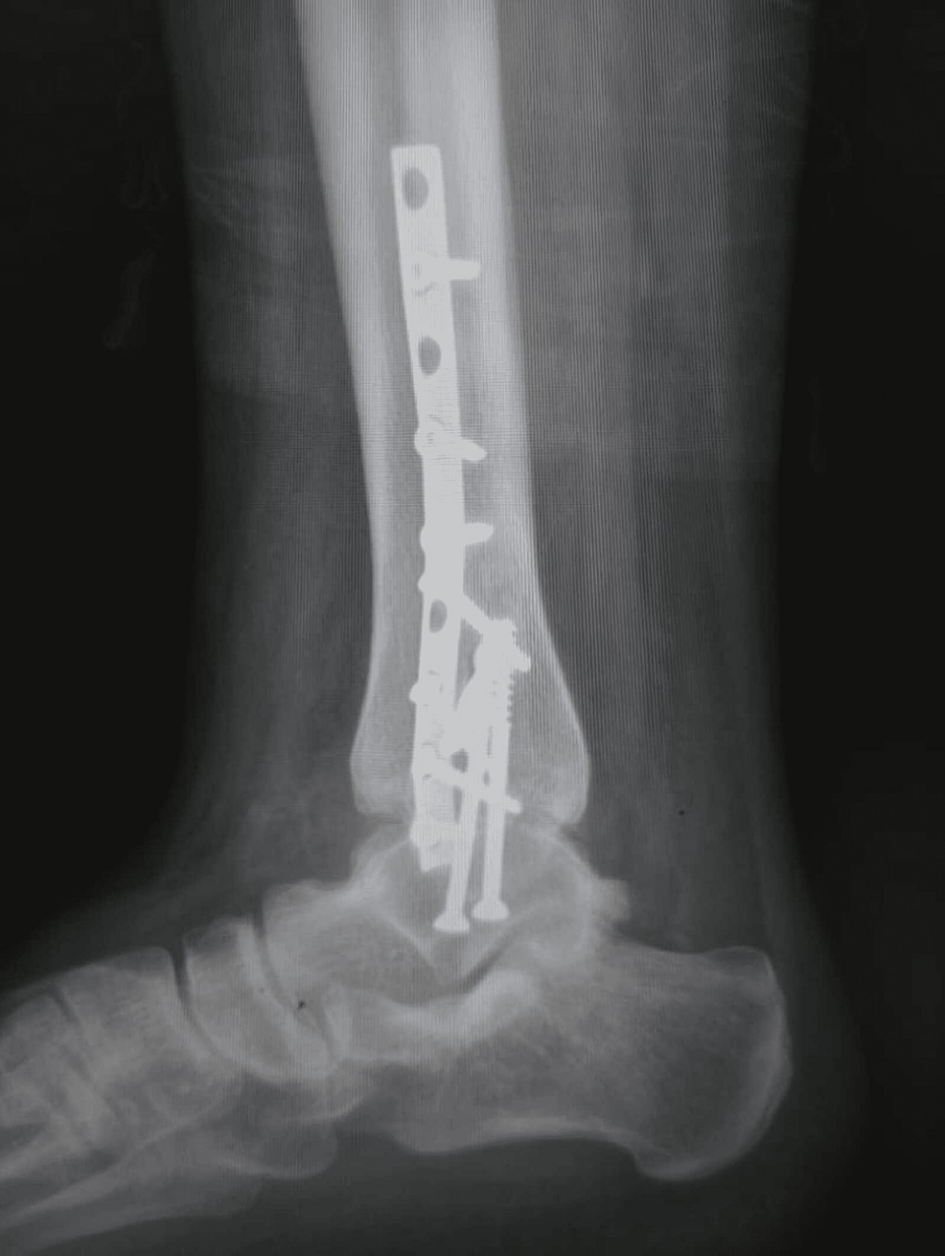
Follow-up radiograph lateral view

Follow-up radiographs were performed in standing weight-bearing anteroposterior and lateral views. Radiographic assessment for quality of reduction with measurement of any displacement, union, and development of joint arthrosis was done. The anatomical reduction was achieved and maintained through the follow-up period. The complete union was achieved three months after the operation without the development of ankle joint arthritis. Plain radiographs after 18 months showed maintained normal alignment without affection of the articular surface (Figures 7-8).

Physiotherapy started at the third postoperative with a supervised program besides home exercises. 

## Discussion

Open anatomical reduction and rigid internal fixation are the treatment of choice in displaced ankle fractures. The time of surgical intervention is usually guided by soft tissue conditions. Soft tissue edema mostly subsides and allows surgical fixation within 10 days from the time of injury. A cohort study by Naumann et al. on 1011 patients who underwent open reduction and internal fixation described the functional outcomes after 3-6 years; they reported that there is no clear association between the time of surgical intervention and postoperative complications. Moreover, maybe there is a safe phase of surgical fixation up to six days after the onset of injury [11]. There is no agreed definition now of the term delayed surgical fixation of the ankle, and there is no precise time after which surgical intervention is contraindicated or associated with an unfavorable outcome.

Fogel and Morrey treated 26 ankle fractures with delayed surgical intervention between 14-31 days after the initial injury; they defined the upper limits for delayed open reduction and internal fixation by 31 days from the time of injury [12].Chiu et al. reported 13 cases of surgical intervention for ankle fractures two to 36 months after the injury [13]. They found improvement in all cases, whatever the time of surgical fixation, but patients who operated less than six months from injury had better outcomes [12-13].

There are few **c**ases of ankle fracture with delayed presentation, which makes it difficult to set guidelines and manage accordingly. Management of neglected cases of ankle fracture is individualized according to the pattern of fracture, syndesmotic injury, talar injury, associated articular cartilage damage, ligaments injury, radiological signs of arthrosis, age at the time of presentation, sex, occupation, patient expectation, and lifestyle.

Neglected cases of ankle fracture usually need more workup of radiological assessment, which will help proper preoperative planning. Surgical techniques are usually complex in neglected cases to avoid intraoperative complications and do proper reduction to have a functional congruent joint. 

According to the Arbeitsgemeinschaft für Osteosynthesefragen/Association for the Study of Internal Fixation (AO) philosophy, intra-articular fracture management should be anatomical reduction and rigid internal fixation [14]. In acute cases of ankle fracture, the key to successful fixation results is anatomical reduction; also, favorable outcomes in neglected cases of ankle fracture depend mainly on good quality reduction [13]. Poor outcomes are usually due to poor surgical techniques and bad quality reduction [13,14].

Arthritic changes of the ankle may affect up to 4% of the population, 70% of them due to ankle fractures [15-16]. Osteoarthritic changes following ankle fracture surgical management are still high, and articular surface involvement will accelerate the arthritic changes [17]. 

The main risk components for the development of arthritis include joint cartilage involvement, ankle instability, incongruent joint, and abnormal alignment [18]. Surgical treatment aims to have stable, congruent joint with normal alignment. To a great extent, modification of risk components will delay the onset of arthritis and have a window of a painless joint which is one of the goals of surgical fixation.

## Conclusions

The time of surgical fixation of ankle fractures is very critical in guiding the postoperative outcomes and avoiding complications. If there is no significant joint arthritis, open anatomical reduction with rigid internal fixation is the gold standard treatment in malunited ankle fracture, whenever the time of presentation. A guideline is still needed for the management of cases with delayed presentation or neglected ones.
